# Serine protease inhibitor kazal-type 6 inhibits tumorigenesis of human hepatocellular carcinoma cells via its extracellular action

**DOI:** 10.18632/oncotarget.13983

**Published:** 2016-12-16

**Authors:** Kuikui Ge, Jinjiang Huang, Wei Wang, Meigang Gu, Xinchuan Dai, Yuqiang Xu, Hongyu Wu, Guodong Li, Hairong Lu, Jiang Zhong, Qingshan Huang

**Affiliations:** ^1^ State Key Laboratory of Genetic Engineering, School of Life Sciences, Fudan University, Shanghai 200433, China; ^2^ Laboratory of Virology and Infectious Disease Center for the Study of Hepatitis C, Rockefeller University, New York, NY 10065, USA; ^3^ Shanghai High-Tech United Bio-Technological R&D Co., Ltd, Shanghai 201206, China

**Keywords:** SPINK6, HCC, tumor suppressor, invasion, proliferation

## Abstract

Hepatocellular carcinoma (HCC) causes significant medical burdens worldwide. Diagnosis, especially in the early stages, is still challenging. Therapeutic options are limited and often ineffective. Although several risk factors have been known important for development of HCC, the molecular basis of the process is rather complex and has not been fully understood. We have found that a subpopulation of HCC cells which are resistant to oncolytic parvovirus H1 superinfection highly express serine protease inhibitor Kazal-type 6 (SPINK6). This protein is specifically reduced in all HCC cell lines and tissues we analyzed. When upregulated, SPINK6 could suppress the malignant phenotypes of the HCC cells in several *in vitro* models. The putative tumor suppression role of SPINK6 is, however, independent of its protease inhibitory activity. To suppress the malignancy of HCC cells, SPINK6 has to be secreted to trigger signals which regulate an intracellular signaling molecule, ERK1/2, as well as a series of downstream factors involved in cell cycle progression, apoptosis and migration. Our study supports that SPINK6 is an important tumor suppressor in liver, and further investigations may help develop more effective diagnostic and therapeutic approaches.

## INTRODUCTION

Hepatocellular carcinoma (HCC) is the fifth most prevalent cancer and the third most frequent cause of cancer mortality globally. Each year there are approximately 630,000 new cases of HCC in the world and more than half of the new cases occur in China [1–4]. HCC in its early stages is often asymptomatic. To increase the accuracy of diagnosis, various serological markers, sophisticated imaging modalities and liver biopsy are required [5]. Currently, there is no effective chemotherapy or radiotherapy. Surgical operation is the only opportunity for a long-term cure and can only be accomplished in a limited number of patients [5]. While studies have found that aberrant actions of many gene products are associated with development of HCC, various genetic and environmental factors are known to contribute to the highly heterogeneous pathogenesis of HCC, making it difficult to determine which events are critical in tumor initiation versus progression [6–11]. Studies aimed at clarifying the molecular mechanism of HCC development are therefore highly needed. The results will help improve the diagnostic and therapeutic approaches.

Proteases and their natural inhibitors are often aberrantly regulated to support tumor expansion [12]. Human serine protease inhibitor Kazal-type 6 (SPINK6) has recently been found to be a selective inhibitor of Kallikrein-related peptidases (KLKs) in skin [13]. Its inhibitory activity is likely essential to prevent aberrant KLK-mediated cleavages, which have been linked to many diseases including skin cancer [14, 15]. Like other SPINK family members, SPINK6 contains one conserved Kazal domain with six cysteine residues forming three intra molecular disulfide bridges [16]. Its crosslinking to extracellular fibronectin allows it to act as a specific KLK inhibitor in epidermis [17–19]. It is worth noting that the function of SPINK6 is probably not limited in skin, as SPINK6 mRNA exists in many other tissues including liver [13]. So far, the function of SPINK6 in non-skin tissues is largely unexplored, and it remains unclear whether its inhibitory activity against the KLK peptidases is also important in any of those tissues.

The rodent autonomous oncolytic parvovirus H1 (H-1PV) is a small (~25 nm), non-enveloped virus with a single-stranded DNA genome (~5 kb). It can kill *in vitro*-transformed or tumor-derived human and rodent cells, with no cytopathic effects on normal cells [20]. This selective oncolytic effect of H-1PV was used to efficiently isolate normal cells embedded in tumor tissues [21–23]. Interestingly, it was also used to acquire a subpopulation of tumor cells with reduced malignancy from a leukemia cell line. The follow-up investigation of the molecular basis of their resistance to H-1PV infection revealed the key role of p53 in tumor suppression pathways [24]. Apparently, this approach can be further used to acquire resistant cells from other tumor tissues, such as HCC. As the actions of the underlying molecules responsible for suppressing the malignancy of the resistant cells may be largely dependent on the tissue specific pathways, our application of this approach to HCC may lead to identification of tumor suppressors that predominantly act in liver. The functional loss of those factors may be critical in HCC development.

We challenged an HCC cell line QGY7703 with H-1PV superinfection and indeed acquired a subpopulation of surviving cells with suppressed malignant phenotypes. When comparing gene expression profiles between the original QGY-7703 cells and those resistant to the H-1PV superinfection, we found that SPINK6 is one of the factors highly expressed in the resistant cells, indicating that SPINK6 is a putative tumor suppressor in hepatocytes. Our follow-up study confirmed that SPINK6 inhibits the activation of extracellular-signal-regulated kinases 1/2 (ERK1/2) through its extracellular action, which is independent of its protease inhibitory activity. Overall our study supported the importance of SPINK6 in suppression of HCC, and justified further investigations for the development of more effective SPINK6-based diagnostic and therapeutic tools.

## RESULTS

### Expression of SPINK6 is decreased in human HCC

**Figure 1:**
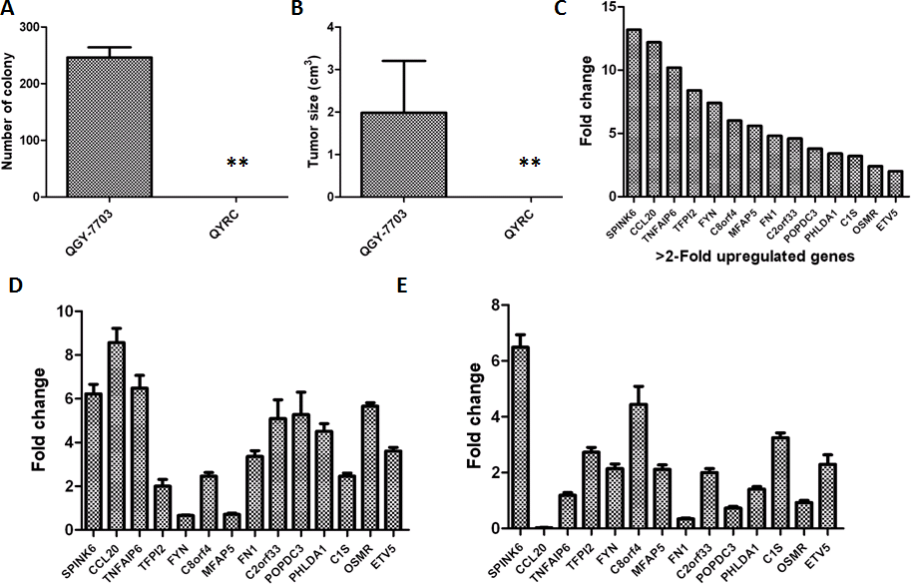
Analysis of an H-1PV resistant QYRC cell line and its gene expression profile (**A**) Colony formation of the HCC cell line QGY-7703 and its derived H-1PV resistant cell line QYRC in soft agar assays. (**B**) Growth and tumor formation of the QGY-7703 and QYRC cells hypodermically injected into nude mice (*n* = 5). Results in (A) and (B) are expressed as the mean ± SD; (**C**) cDNA microarray analysis of gene expression in the QGY-7703 and QYRC cells. Genes with more than 2-fold mRNA upregulation in the QYRC cells are listed in a declining order from left to right. (**D**) RT-qPCR quantitation and comparison of mRNA levels of the selected genes in the QGY-7703 and QYRC cells. The folds of mRNA upregulation in the QYRC cells are plotted. (**E**) RT-qPCR quantitation and comparison of mRNA levels of the selected genes in HCC and normal human hepatic tissues. The folds of mRNA upregulation in normal hepatic tissues are plotted. GAPDH mRNA levels were used as internal controls for RT-qPCR quantitation.

We superinfected a hepatocellular carcinoma (HCC) cell line (QGY-7703) with oncolytic parvovirus H1 (H-1PV) and acquired a group of resistant cells QYRC. Previous studies suggested that cells surviving H-1PV superinfection should behave somewhat similarly to normal cells [25]. When compared with the QGY-7703 cells, the QYRC cells indeed exhibited loss of colony formation in soft agar (Figure 1A), and they didn't form detectable tumors after being hypodermically injected into 5 nude mice (Figure 1B). In order to find out what cellular factors may be responsible for the loss of tumor cell phenotype, we compared the cDNA microarray profiles of the QGY-7703 and QYRC cells. We found that a series of genes were upregulated in the H-1PV-resistant QYRC cells (Figure 1C). To confirm the upregulation of those genes, we used the more accurate real-time PCR approach to compare the mRNA levels of those genes in the QYRC and QGY-7703 cells. Indeed, they were all upregulated, although the relative folds of changes were different from those in the microarray analysis (Figure 1D). We also analyzed those mRNAs extracted from hepatocarcinoma and the adjacent normal tissues, and found that most of them were expressed at higher levels in normal tissues (Figure 1E). It is worth noting that we consistently detected high expression of SPINK6 in both QYRC and normal tissues, suggesting that SPINK6 is an important factor associated with normal liver cells (Figure 1C–1E).

**Figure 2:**
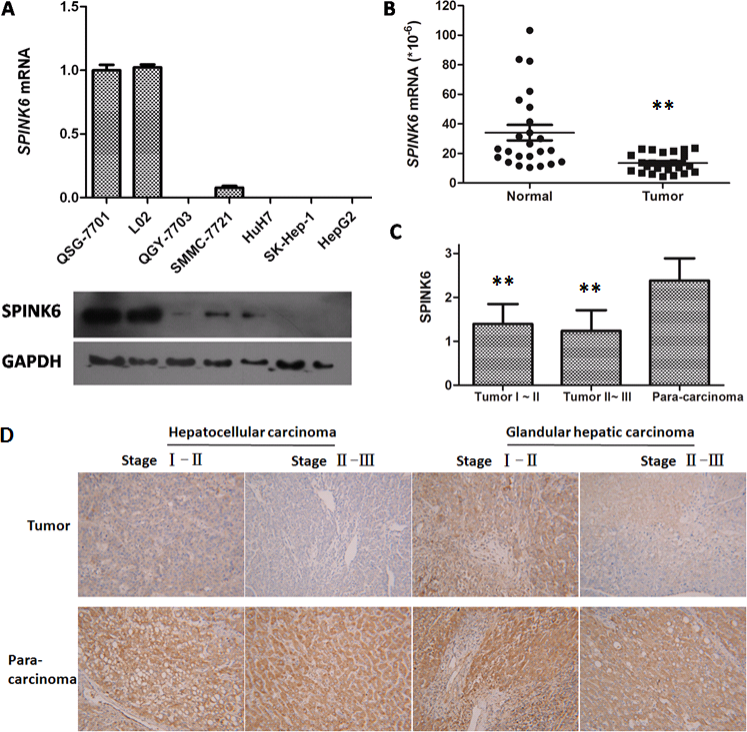
Expression of SPINK6 is reduced in HCC cell lines and tissues (**A**) Comparison of SPINK6 mRNA and protein levels between normal liver cells (QSG-7701 and L02) and HCC cells (QGY-7703, SMMC-7721, HuH7, SK-Hep-1, HepG2) by RT-qPCR and western blot. The mRNA quantities from different cell lines are presented as columns. Below each column is the blotted SPINK6 protein from the same cell line. The cell line names are noted in the middle. GAPDH is an internal control. (**B**) Comparison of SPINK6 mRNA levels between HCC and adjacent normal tissues by Liver Cancer Tissue RT-qPCR Array. The marker bars represent statistic averages. **P < 0.01, *n* = 24. (**C**) Comparison of SPINK6 protein levels between tumor and adjacent normal tissues. SPINK6 proteins were immune-stained in 48 HCC tissues and the normal para-carcinoma tissues. The HCC tissues were categorized into two groups, stage I–II and II–III. The staining intensities were quantitated using an Image-Pro Plus6.0 software. The overall staining in tumor tissues is significantly lower than that in the adjacent normal tissues (**P < 0.01, *n* = 48). (**D**) Representative pictures of HCC and adjacent normal tissues immune-stained against SPINK6. The top panels represent stage I–II HCC tissues, stage II–III HCC tissues, stage I–II glandular hepatic carcinoma tissues, and stage II–III glandular hepatic carcinoma tissues. The bottom panels represent the corresponding adjacent normal tissues.

We wondered whether SPINK6 is specifically downregulated during HCC development. We first compared its expression levels in five HCC cell lines (QGY-7703, SMMC-7721, HuH7, SK-Hep-1, HepG2) and two normal liver cell lines (QSG-7701, L02). We found that both mRNA and protein levels of SPINK6 in all HCC cell lines were lower than those in the normal liver cells (Figure 2A). To understand whether this also happens *in vivo*, we further analyzed SPINK6 levels in a commercial cDNA array prepared from both hepatocarcinoma and normal human liver tissues. The SPINK6 mRNA levels were overall lower in the hepatocarcinoma tissues than in the normal liver tissues (13.52 ± 1.30 and 34.09 ± 5.30, respectively; *P* < 0.001) (Figure 2B). We also determined the protein levels of SPINK6 by immunostaining hepatocarcinoma tissues and the matched para-carcinoma normal tissues in a tissue microarray consisting of 48 patient samples (Figure 2C and 2D). Quantitation of the immunostaining results confirmed that the SPINK6 protein levels were reduced in all tumor tissues grouped as period I–II and II–III (1.40 ± 0.45 and 1.24 ± 0.47, respectively), while those in the adjacent normal tissues were relatively high (2.38 ± 0.51) (Figure 2C). Notably, SPINK6 expression was nearly undetectable in tumor tissues of advanced stages (Figure 2D). Together, these results strongly suggested that SPINK6 may be a tumor suppressor and its expression may be reduced as HCC develops.

### SPINK6 inhibits the proliferation, migratory and tumorigenic abilities of HCC cells

**Figure 3:**
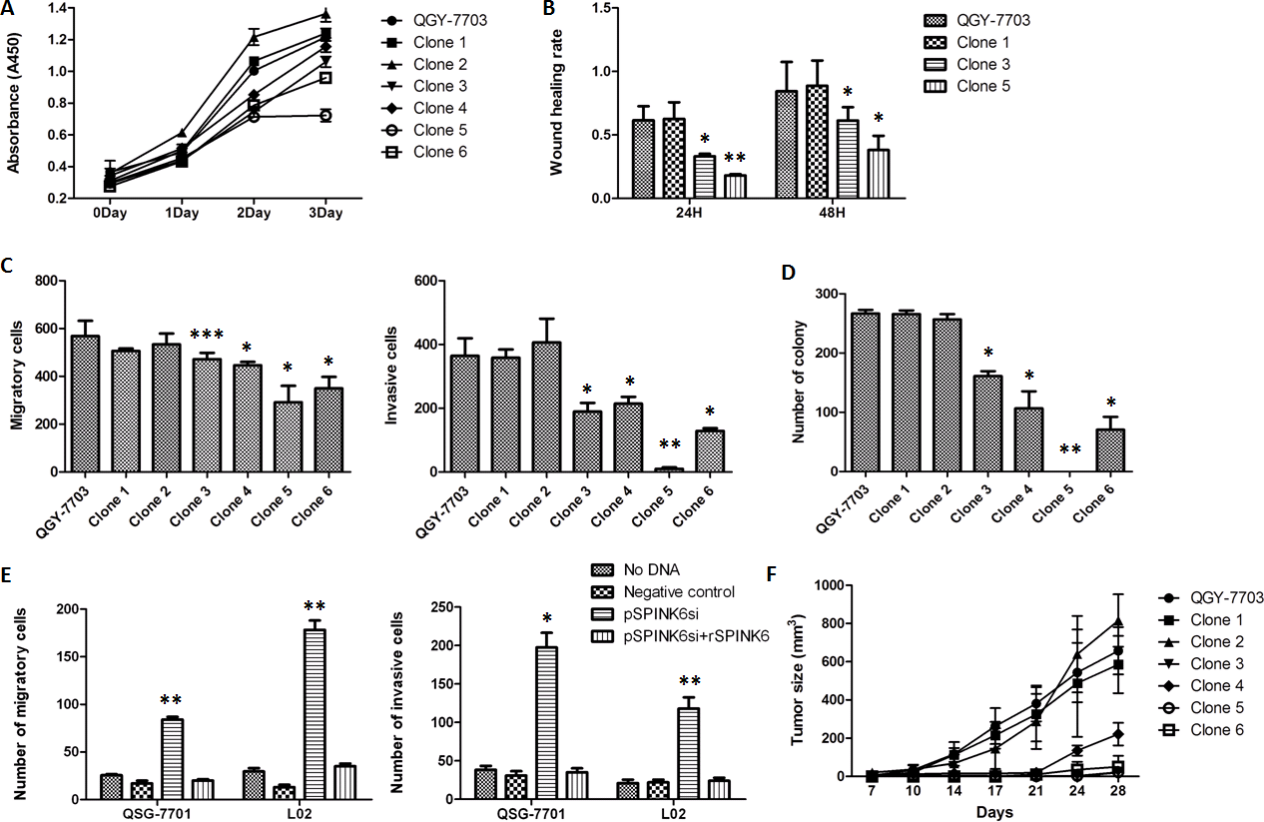
SPINK6 can suppress the malignancy phenotypes of QGY7703 HCC cells (**A**) Growth of the cell clones expressing SPINK6 at different levels. The QGY-7703 HCC cells were transfected with plasmids carrying the SPINK6 gene, and the isolated clones express SPINK6 to relatively low (clone 1 and 2), medium (clone 3 and 4) and high levels (clone 5 and 6). (**B**) Wound healing assays on the isolated cell clones 1, 3 and 5 at 24 and 48 h. (**C** and **D**) Migration, invasion and colony formation of QGY-7703 and the derived the cell clones. (**E**) Silencing of SPINK6 enhanced migration and invasion of normal liver cells (QSG-7701 and L02). Normal liver cells were transfected with plasmids expressing siRNA targeting SPINK6 mRNA (pSPINK6si) or an empty vector (negative control). Recombinant SPINK6 (rSPINK6) was added into culture medium at a 50 ug/ml concentration. (**F**) Growth and tumor formation of QGY-7703 and the derived cell clones hypodermically injected into nude mice. The data points in all panels were averaged from at least 3 assay replicates. The error bars represent standard deviation. *P < 0.05, **P < 0.01, ***P < 0.001.

In order to quantitatively probe the impact of SPINK6 expression on hapatocarcinogenesis, we generated a panel of cell lines expressing different levels of SPINK6 and compared their tumorigenic phenotypes. We transfected the QGY-7703 cells with vectors carrying the SPINK6 gene and isolated clones expressing SPINK6 to relatively low (clone 1 and 2), medium (clone 3 and 4) and high levels (clone 5 and 6) (Supplementary Figure S1A). The cells with medium to high-level expressions of SPINK6 showed slower proliferation than the original QGY-7703 cells, and the higher expression seemed to be associated with stronger reduction in proliferation rates (Figure 3A). When tested in wound healing assays at both 24 and 48 hour time points, the cells derived from clones 3 and 5 with medium to high-level expressions of SPINK6 reduced migration, and there was an apparently correlation between the expression level of SPINK6 and the extent of reduction in the wound healing rates (Figure 3B). Further analysis of all cell clones with transwell assays and colony formation with soft agar assays also revealed that the medium to high-level expression of SPINK6 were associated with reduced cell migration, invasion and colony formation (Figure 3C and 3D).

We also tried to examine the impact of loss of SPINK6 in the “normal” liver cells (QSG-7701 and L02). We constructed a SPINK6-specific siRNA vector (siSPINK6). Transfection of siSPINK6 effectively downregulated the expression of SPINK6 in the QSG-7701 and L02 cells as determined by RT-PCR and western blot (Supplementary Figure S1B and S1C). Downregulation of SPINK6 apparently enhanced the tumorigenic phenotypes of the cells as indicated by the increased number of migrating and invading cells (Figure 3E). The normal cell phenotypes could be rescued, however, when recombinant SPINK6 (rSPINK6) was introduced into the culture medium, supporting a role for SPINK6 in suppressing tumorigenesis of liver cells (characterization of the recombinant SPINK6 protein will be further discussed below.).

Given the results from cultured cells, we tried to examine whether formation and growth of tumors from implanted hepatocarcinoma cells would be impacted by overexpression of SPINK6 in those cells. We implanted the QGY-7703 cells and all SPINK6 expressing cells derived from clones 1–6 into nude mice by hypodermic injection, and monitored the appearance of tumors at the sites of injection over 28 days. The tumors derived from the injected clones 3–6 were significantly smaller than those from the cells (QGY-7703, and clones 1 and 2) with low-level SPINK6 (Figure 3F). The results strongly support the importance of SPINK6 in suppressing tumor formation from injected HCC cells.

To find out whether SPINK6 can inhibit the tumorigenic phenotypes of other hepatocarcinoma cells, we also overexpressed SPINK6 in three HCC cell lines HepG2, HuH7 and SK-Hep-1 (Supplementary Figure S1D and S1E). As expected, overexpression of SPINK6 reduced cell migration, invasion and proliferation of all HCC cell lines, and significantly reduced tumor growth when the HuH7 and SK-Hep-1 cells were implanted into nude mice (Supplementary Figure S2).

### SPINK6 is secreted to inhibit proliferation of HCC

**Figure 4:**
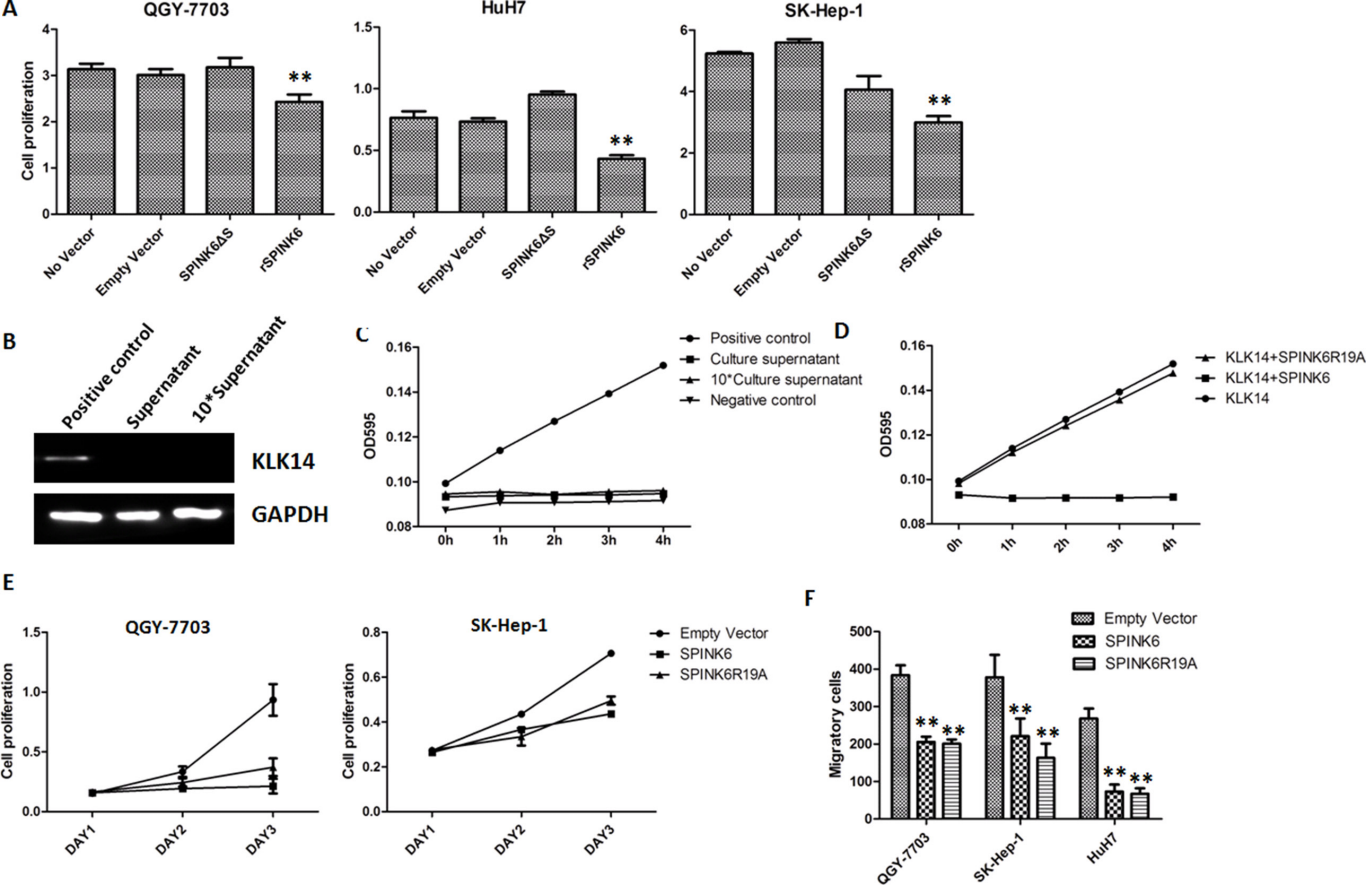
The function of SPINK6 requires its secretion into extracellular space but is independent of its KLK14 inhibitory activity (**A**) Proliferation of HCC cells (QGY-7703, HuH7 and SK-Hep-1). Cells were transfected with a plasmid expressing SPINK6 without the signal peptide (SPINK6DS), or supplemented with recombinant SPINK6 (rSPINK6) in culture medium. No vector and empty vector transfection are negative controls. (**B**) Western blot analysis of KLK14 in cultural supernatant of hepatocellular carcinoma cells QGY-7703. Cell lysis was used as positive control. (**C**) Activity analysis of KLK14 in cultural supernatant of hepatocellular carcinoma cells QGY-7703. Recombinant KLK14 protein (rKLK14) and culture medium were used as positive and negative controls respectively. (**D**) Activity analysis of KLK14 in the presence of inhibitory rSPINK6 or a non-inhibitory mutant rSPINK6R19A. (**E**) Proliferation of HCC cell lines (QGY-7703 and SK-Hep-1) decreased with overexpression of SPINK6 or SPINK6R19A. *n* = 4. (**F**) Migration and invasion of HCC cell lines (QGY-7703, HuH7 and SK-Hep-1) decreased with overexpression of SPINK6 or SPINK6R19A. The data points were averaged from at least 3 assay replicates. The error bars are standard deviation. *P < 0.05, **P < 0.01, ***P < 0.001.

Analysis of the coding sequence of SPINK6 revealed that the protein contains a signal peptide, which should direct the newly translated proteins to the secretory pathway. We wondered whether secretion is required for SPINK6 to suppress the tumorigenic phenotypes of the HCC cells. We therefore expressed SPINK6 without the signal peptide in the HCC cell lines (Supplementary Figure S3), and found that overexpression of the truncated protein could not suppress proliferation of the QGY-7703, HuH7 and SK-Hep-1 cells. Cell proliferation was, however, inhibited when the recombinant SPINK6 protein was added into cell culture medium (Figure 4A), suggesting that the extracellular presence of SPINK6 is critical for its potential anti-proliferative function.

### Inhibition of the KLK proteases is not required for SPINK6-mediated tumor suppression

SPINK6 has been reported as a potent inhibitor of epidermal Kallikrein-relatedproteases (KLKs), including KLK5, KLK7 and KLK14, the abnormal expressions of which have been implicated in carcinogenesis. The inhibitory target of SPINK6 in liver, however, has not been described. The apparent lack of KLK14 in the cultural supernatant of hepatocellular carcinoma cells suggested that inhibition of KLK14 is not involved in SPINK6 mediated suppression of tumorigenic phenotypes (Figure 4B and 4C). In order to find out whether inhibition of any other KLK proteases is involved, we engineered a SPINK6 mutant SPINK6R19A, which has the critical residue substituted and cannot inhibit any KLK protease (Figure 4D). Interestingly, this mutant acted like the wild type protein, suppressing the proliferation, migration and invasion of different lines of HCC cells (Figure 4E and 4F). The results suggested that the potential anti-tumor activity of SPINK6 does not require its inhibition of the KLK proteases.

### SPINK6 triggers regulations upon ERK1/2

Our results have shown that SPINK6 is secreted into the extracellular space, where it may trigger some tumor suppressing signaling pathways to suppress the tumorigenic phenotypes of the HCC cells. To understand the molecular mechanism of SPINK6-triggered tumor suppression, we analyzed the impact of SPINK6 overexpression on different signaling pathways linked to carcinogenesis in HCC cells.

**Figure 5:**
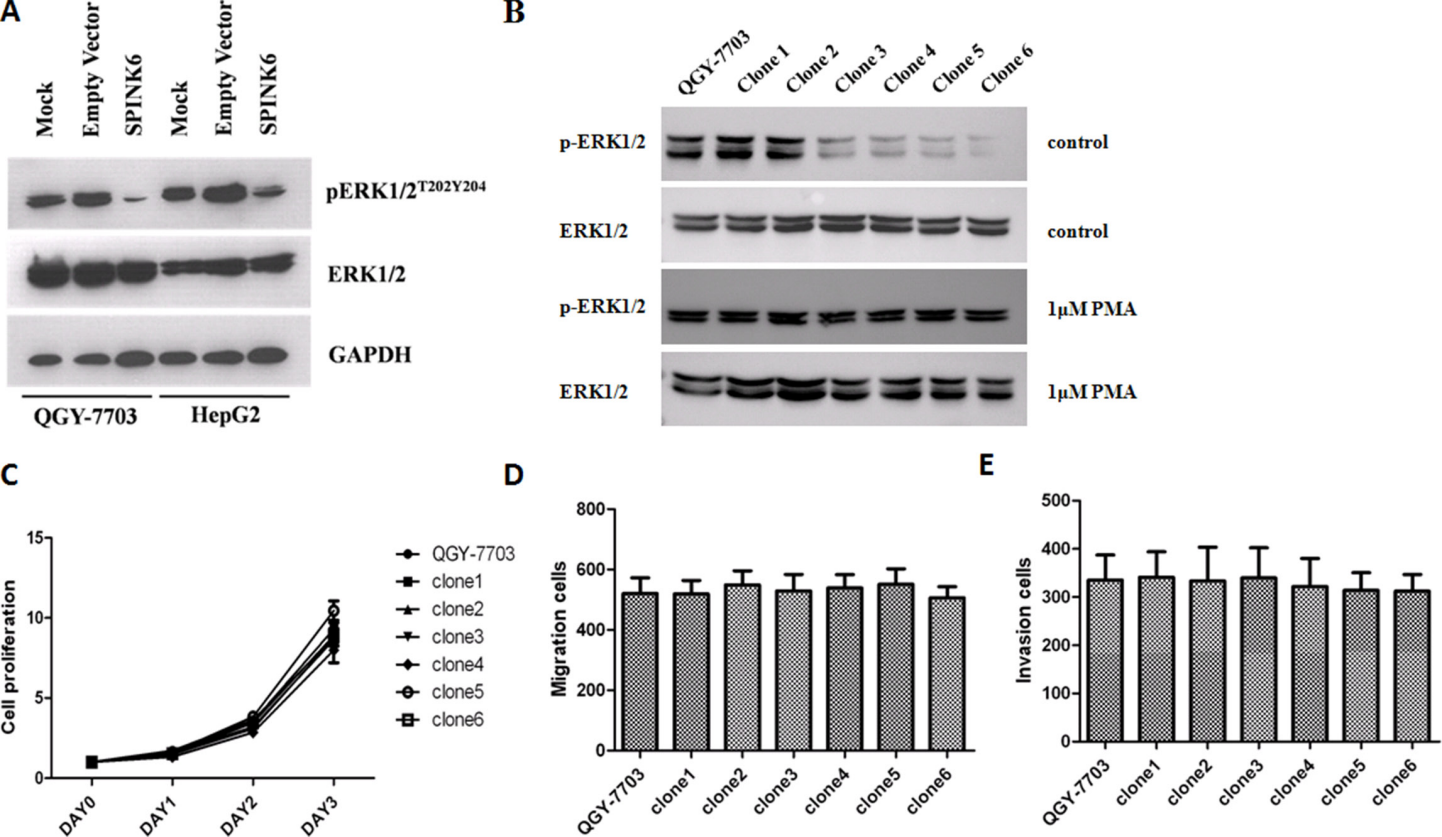
SPINK6 inhibits ERK1/2 phosphorylation (**A**) Western blot analysis of phosporylated (pERK1/2^T202Y204^) and total ERK1/2. Cells (QGY-7703 and HepG2) were transfected with a SPINK6-expressing vector or an empty vector. Mock represents untransfected cells. GAPDH is an internal blotting control. (**B**) Western blot analysis of phosphorylated ERK1/2 in QGY-7703 and the derived cell clones expressing increasing amounts of SPINK6. (**C**–**E**) The proliferation, migration and invasion of QGY-7703 cell clones. The PMA compound was added at a 1 μM concentration to enhance ERK1/2 action continuously. The data points were averaged from 3 assay replicates. Results are expressed as the mean ± SD;

Extracellular-signal-regulated kinases (ERK1/2) are often aberrantly activated by phosphorylation in response to external and internal stimuli in different types of tumors [26, 27]. We found that over-expression of SPINK6 in the HCC cell lines QGY-7703 and HepG2 remarkably inhibited the phosphorylated species of ERK1/2, while the total protein levels were not significantly changed (Figure 5A). The stronger reduction of ERK1/2 phosphorylation seems to be correlated with higher expression of SPINK6 (Figure 5B). To further confirm that the potential tumor suppression role of SPINK6 requires ERK1/2, we incubated the cells with excess PMA which could excite continuous phosphorylation of ERK1/2 beforehand and then tested whether SPINK6 could still suppress the tumorigenic phenotypes. In the presence of 1μM PMA, the phosphorylation of ERK1/2 of different clones were indistinctive, meanwhile the tumor suppression function of SPINK6 seemed to be blocked (Figure 5C–5E). Similar results were showed in present in 10μM U0126 which could inhibit phosphorylation of ERK1/2, the phosphorylation of ERK1/2 of different clones were also indistinctive, the overexpression of SPINK6 could not reduce the phosphorylation of ERK1/2 more, so the tumor suppression function of SPINK6 seemed to be also largely blocked, although minor inhibition of the tumorigenic phenotypes could be still detected (Supplementary Figure S4). This strongly supported that ERK1/2 is required for SPINK6 function. We did not detect significant changes of JNK, p38 or other signaling proteins (Supplementary Figure S5). Overall, we believe that SPINK6 mainly inhibits ERK1/2 activation, although at this stage we cannot rule out the possibility that it may render broader regulation through other signaling molecules.

### Regulations upon ERK1/2 affect several factors implicated in tumor development

**Figure 6:**
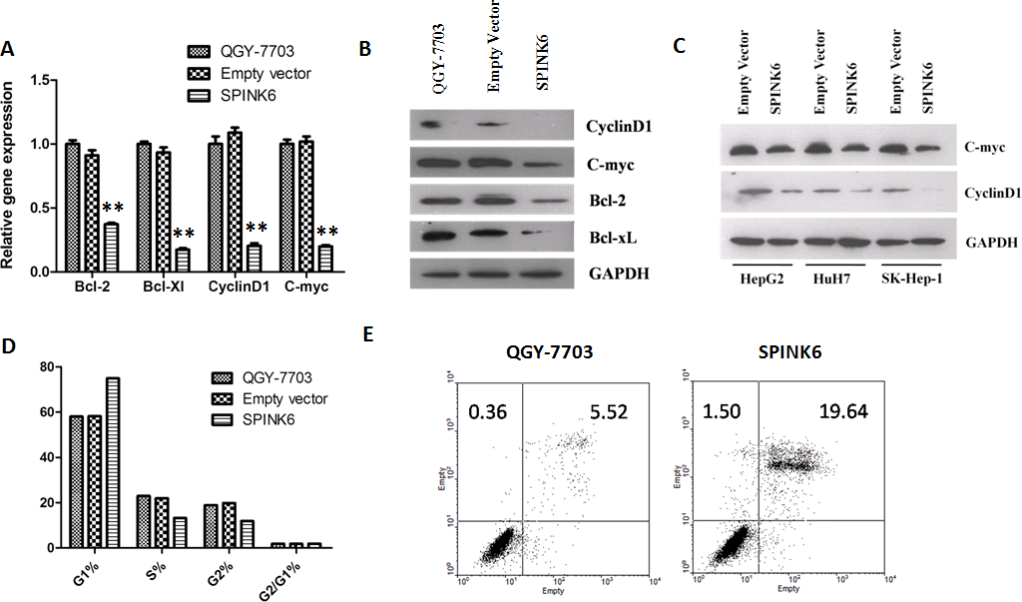
SPINK6 expression downregulates ERK1/2 downstream factors (**A**) RT-PCR quantitation of the mRNA levels of cyclinD1, c-myc, Bcl-2 and Bcl-xL. (**B**) Western blot analysis of cyclinD1, c-myc, Bcl-2 and Bcl-xL. (**C**) Western blot analysis of CyclinD1 and c-myc in HCC cells (HuH7, SK-Hep-1, and HepG2) transfected with either an empty vector or a SPINK6-expressing vector. GAPDH is as an internal control in (A–C). (**D**) Percentage of cells at different stage of cell cycle. The cells were transfected with either an empty vector or a SPINK6-expressing vector in (A–D). The untransfected QGY-7703 cells in (A), (B) and (D) are mock controls. (**E**) Flow cytometry analysis of cell apoptosis. The QGY-7703 cells and those transfected with a SPINK6 expressing vector were treated with 1 μM Cisplatin.

We predicted that SPINK6-mediated inhibition of ERK1/2 phosphorylation would probably impact some of the downstream targets. We found that at least four intracellular factors, c-Myc, cyclin D1, Bcl-2 and Bcl-xL were downregulated at the protein level when SPINK6 was expressed in the HCC cells (Figure 6A to 6C). c-Myc is a transcriptional factor involved in cell cycle progression, apoptosis and cellular transformation. Cyclin D1 is an important cell cycle regulator [28, 29]. Bcl-2 and Bcl-xL are two apoptosis suppressors [30, 31] . The reduced levels of these factors may explain the suppressed tumorigenic phenotypes of the HCC cells. Indeed, we found that the SPINK6 expressing HCC cells seemed to experience longer G1 phase, and more cells died through apoptosis when treated with cisplatin, a chemotherapeutic reagent which triggers DNA damage (Figure 6D and 6E). We also checked the protein levels of several extracellular factors, which can be affected by ERK1/2 and has been linked to tumor development. We found that a serine protease uPA (urokinase-type plasminogen activator) and its receptor uPAR, and two matrix metallopeptidases MMP9 and MMP20, were downregulated upon SPINK6 expression in the HCC cells and result of gelatin zymography also showed that the biological activity of MMP9 was inhibited during the over-expression of SPINK6 (Supplementary Figure S6). These factors have been suggested to play important roles in proteolysis of extracellular matrix (ECM), facilitating cell migration [12, 32]. This may explain the reduced migration of the HCC cells expressing high levels of SPINK6.

## DISCUSSION

Many important proteins are aberrantly expressed in the process of tumor development and thus can be identified by comparing gene expression profiles between tumor and normal cells. Although many cell lines appear homogenous by morphology or culture characteristics, they are rather heterogeneous at the molecular level. The aberrant expression of some important factors may be limited to a subpopulation of the mixed cells, and the overall expression levels of those factors may only show small changes in a large scale transcriptome comparison between cell lines. The potential HCC suppressor SPINK6 is probably only well expressed in some types of cells mixed in the original QGY-7703 cell line. We likely enriched those SPINK6 high expressing cells by acquiring the H-1PV resistant cells. The follow up comparison of the gene expression profiles between the H-1PV resistant cells and the original QGY-7703 cells indeed helped us find SPINK6 as one of the most differentially expressed genes between those two types of cells. This approach may be further used to analyze other types of tumor cells, facilitating identification of new cellular factors involved in regulation of tumorigenesis.

The development of cancer is a consequence of dysregulation of a complex network of biological activities. It has been well accepted that the process of carcinogenesis often manipulates a series of proteases and their natural inhibitors from both tumor cells and non-neoplastic neighboring cells to support tumor expansion [12]. Our study has shown that SPINK6 can suppress tumorigenic phenotypes of liver cancer cells in several *in vitro* models. Although its selective inhibitory activity against several KLK proteases has been thought important in maintaining skin homeostasis, its potential tumor suppression function in liver cells seems to be independent of the residues required to inhibit the KLK members. To suppress the tumorigenic phenotypes of HCC cells, SPINK6 has to be secreted into extracellular space, where it likely triggers regulation of signaling pathways, affecting at least an intracellular signaling molecules, ERK1/2, the regulated activity of which probably further suppresses a series of intracellular and extracellular factors implicated in tumorigenesis.

The signaling pathways mediated by ERK1/2 play a central role in the regulation of various biological processes such as proliferation, survival or cell motility. Aberrant activation of ERK1/2 is frequently observed in human HCC (Guégan, Frémin and Baffet 2012). Small molecules targeting ERK1/2 have been shown effective in blocking proliferation of several tumor models, including HCC [33]. Our study suggests that the potential anti-tumor activity of SPINK6 inhibits ERK1/2 phosporylation. This justifies further investigation of the molecular mechanism of SPINK6-triggered inhibition upon ERK1/2, as identification of molecules playing roles in this context should allow development of new drugs to more effectively suppress the aberrantly activated ERK1/2 in tumors.

Our study showed that the expression of SPINK6 is specifically suppressed in liver tumor tissues. The suppression could be even detected in the early stage tumors. As SPINK6 is a secretory protein, the extracellular protein level may be decreased because of tumor development. Our study therefore justifies further analysis of the correlation between secreted SPINK6 activity and tumor development in HCC patients. The results may help develop new diagnostic tools for detection of early stage HCC development. We also found that recombinant human SPINK6 (rhSPINK6) expressed as a secretory protein from yeast [16] could inhibit the tumorigenic phenotypes of HCC cells when the protein was added into culture medium. This may allow us to utilize the recombinant protein to help treat liver cancer, although further characterization is required to find out whether there is any pathological impact associated with delivery of rhSPINK6 and whether it indeed can suppress tumor growth *in vivo*.

## MATERIALS AND METHODS

### Cell culture

L02, QSG-7701, QGY-7703 and SMMC-7721 cells were cultured in RPMI-1640 supplemented with 10% fetal bovine serum (FBS) and 100 units/ml streptomycin/penicillin. HepG2, HuH7, and SK-Hep-1 cells were cultured in DMEM supplemented with 10% FBS (Life technologies, Carlsbad, CA). The cells were incubated at 37°C in a humidified atmosphere containing 5% CO_2_. All cell lines were purchased from SIBS (Shanghai Institutes for Biological Sciences) in China.

### Animals

BALB/c male nude mice were housed under standard conditions. Animal experiments were performed in agreement with the SIBS Guide for the Care and Use of Laboratory Animals and were approved by the SIBS Animal Care and Use Committee.

### RT-PCR and quantitative real-time PCR

Total RNA for RT-PCR was extracted with TRIZOL (Life technologies, Carlsbad, CA) and was reverse transcribed with the Superscript First-Strand Synthesis System (Life technologies, Carlsbad, CA). Primers for RT-PCR are: *SPINK*6, forward (5′-ATG AAA CTG TCA GGC ATG-3′) and reverse (5′-TCA GCA TTT TCC AGG ATG-3′); *GAPDH*, forward (5′-GCA CCG TCA AGG CTG AGA AC-3′) and reverse (5′-ATG GTG GTG AAG ACG CCA GT-3′).

The mRNA expression of SPINK6 in liver cancer tissues were analyzed by TissueScan Liver Cancer Tissue qPCR Arrays (Origene Technologies, Rockville).

### Cell proliferation assay

Cells were seeded in 96-well plates, and their proliferation was determined by a Cell Counting Kit (Dojindo). Briefly, 10 μl of CCK8 was added to each well (100 μl medium) for 2 hour incubation, followed by measurement of the absorbance at 450 nm in a microplate reader (Thermo Scientific Multiskan MK3). The proliferation of each type of cell was measured four times.

### Antibodies and western blotting analysis

Protein extracts were prepared by lysis in a buffer containing 50 mM Tris-HCl (pH 7.4), 1% NP-40, 5 mM EDTA, 150 mM NaCl, 50 mM NaF, 0.1 mM Na_3_VO_4_, and protease inhibitor (Roche). The proteins were separated by SDS-PAGE (sodium dodecyl sulfate polyacrylamide gel electrophoresis) and transferred to a nitrocellulose membrane. Each target protein was stained by its specific primary antibody which was later detected by a horseradish peroxidase-conjugated secondary antibody raised in a different species. The antibodies we used in this work are anti-SPINK6 (Santa Cruz), anti-GAPDH (Epitomics), anti-ERK, anti-phospho-ERK, anti-c-myc, anti-cyclinD1, anti-Bcl-2 and anti-Bcl-xL primary antibodies, and anti-rabbit horseradish peroxidase-conjugated secondary antibodies (Cell Signaling Technology).

### Immunohistochemistry

A tumor tissue sample array containing 48 liver tumor and adjacent normal tissues was purchased from SuperBiotek. SuperBiotek also performed immunohistochemistry and data quantification. The anti-SPINK6 antibody (Santa Cruz, sc-139065) was used for immunohistochemistry staining.

### Vector construction and transfection

Full-length and truncated *SPINK*6 cDNA without the signal peptide sequence were obtained from a cellular RNA prep by RT-PCR and inserted in a pcDNA3.1(+) vector (Invitrogen). pGPU/GFP/Neo-SPINK6 containing a siRNA sequence targeting the nucleotides 259–279 in the coding sequence of *SPINK6* was purchased from Shanghai GenePharma Co., Ltd .

The plasmids were transfected into target cells using Lipofectamine 2000 (Life technologies). Cells harboring integrated plasmid DNA were selected in the presence of 600 μg/ml of G418.

### Soft agar clonogenic assays

Anchorage-independent growth of the stably transfected cells was examined by colony formation in soft agar. A total of 3 × 10^3^ cells were suspended in 0.3% agar and immediately overlaid on Petri dishes precast with 0.6% agar. Colonies were stained with 1% neutral red and scored after 3-week incubation at 37°C in 5% CO_2_ air. The assays were performed in triplicates.

### Tumorigenesis in nude mice

Male BALB/c nude mice of 4- to 5-week age were used for the xenograft experiments. A total of 2 × 10^6^ cells were injected subcutaneously at a single site in nude mice. Each type of cell was injected into 5 nude mice. The volumes of the tumors formed were measured twice a week using a caliper and calculated as (tumor length × width × height)/2.

### Invasion and migration assays

Standard 24-well chambers (Corning Costar) were used to assess cell migration and invasion following the manufacturer's protocol. Briefly, cells were trypsinized, rinsed twice with PBS, resuspended in serum-free media, and seeded at 1 × 10^5^ cells per well. Chambers in triplicate were placed in growth media as a chemo-attractant. Following 24 h incubation for the migration assay and 48 h incubation for the invasion assay, chambers were fixed in 10% formalin, stained with crystal violet for manual counting.

### Wound healing assays

Cells were seeded into a six-well plate and allowed to grow to 90% confluency. Cell monolayers were wounded with a plastic tip (1 mm), and further cultured in medium supplemented with 10% FBS for 48 h. Cell migration into the wound surface was monitored by microscopy at several time points. The areas of wound heal were analyzed by image pro plus 6.0.

### KLK14 protease activity assay

Cells were seeded into a six-well plate and allowed to grow to 90% confluency, and further cultured in serum free medium for 24 h. The cultural supernatant was collected and concentrated by centrifugal filter devices (Millipore). The activity of KLK14 was determined by measuring chromogenic substrate release by these proteases, as previously described [16]. The protease activity was detected by measurement of the absorbance at 595 nm in a microplate reader (Thermo Scientific Multiskan MK3)

### Statistical analysis

Experiments were repeated for at least three times and data were expressed as mean ± standard deviation and compared between groups using the Student's *t-test*. *P value* of < 0.05 was considered statistically significant.

## SUPPLEMENTARY MATERIALS FIGURES AND TABLES


