# Supplementary material for: Serine protease inhibitor kazal-type 6 inhibits tumorigenesis of human hepatocellular carcinoma cells via its extracellular action
Source: Oncotarget. 2016 Dec 16;8(4):5965–75. doi: 10.18632/oncotarget.13983 (PMC5351605; doi:10.18632/oncotarget.13983)
Supplement: Supplementary file 1 [file oncotarget-08-5965-s001.pdf]

Serine protease inhibitor kazal-type 6 inhibits tumorigenesis of human hepatocellular carcinoma cells via its extracellular action

Supplementary Materials

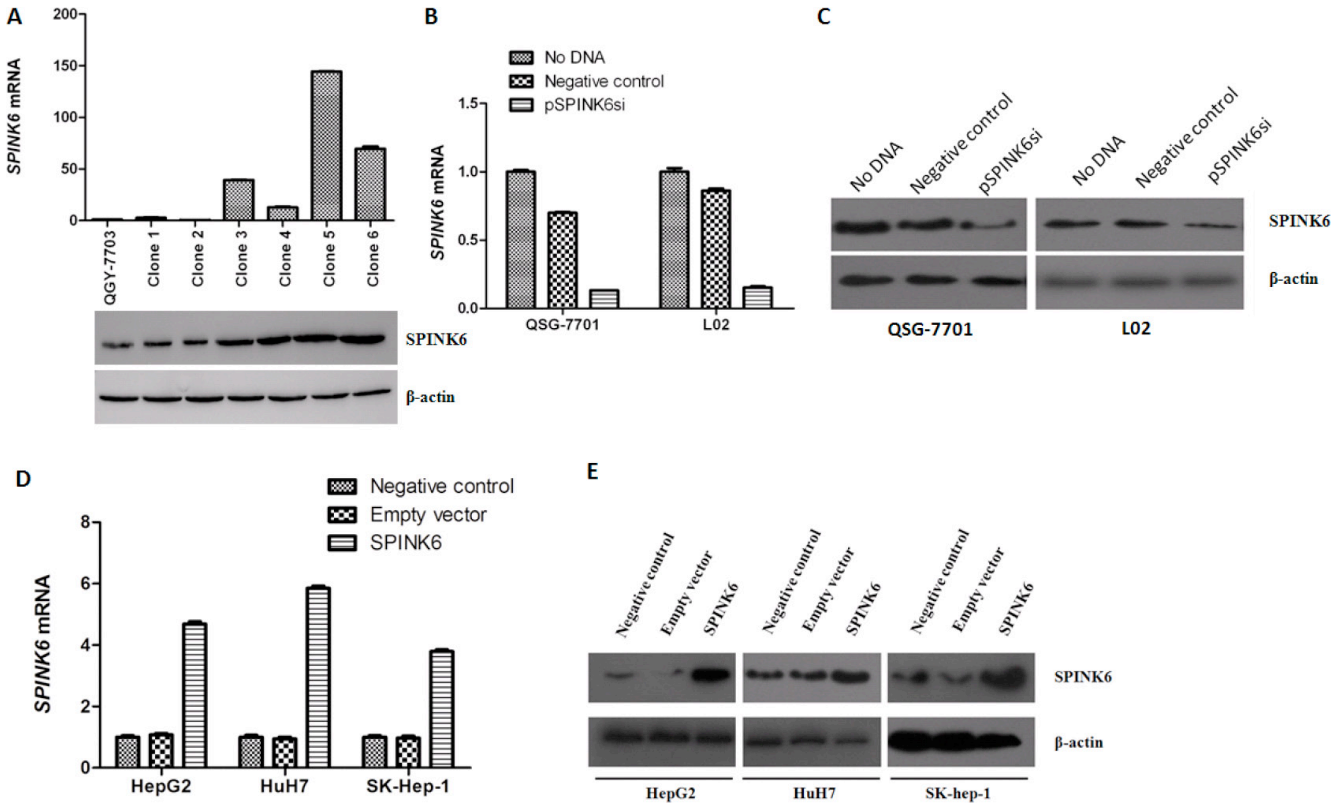

Supplementary Figure S1: Expression of SPINK6 in HCC. (**A**) RT-PCR and western blot analysis of SPINK6 expression in QGY-7703 and its derived cell clones. GAPDH and β-actin are internal controls for RT-PCR and western blot respectively. (**B**, **C**) SPINK6 was efficiently silenced by transfecting siSPINK6, as shown by SPINK6 mRNA and protein levels determined by RT-PCR and western blot respectively. (**D**, **E**) RT-PCR and western blot analysis of SPINK6 mRNA and protein expression in SPINK6-transfected HCC cell lines QGY-7703, HuH7 and SK-Hep-1. GAPDH and β-actin were used as internal controls, respectively.

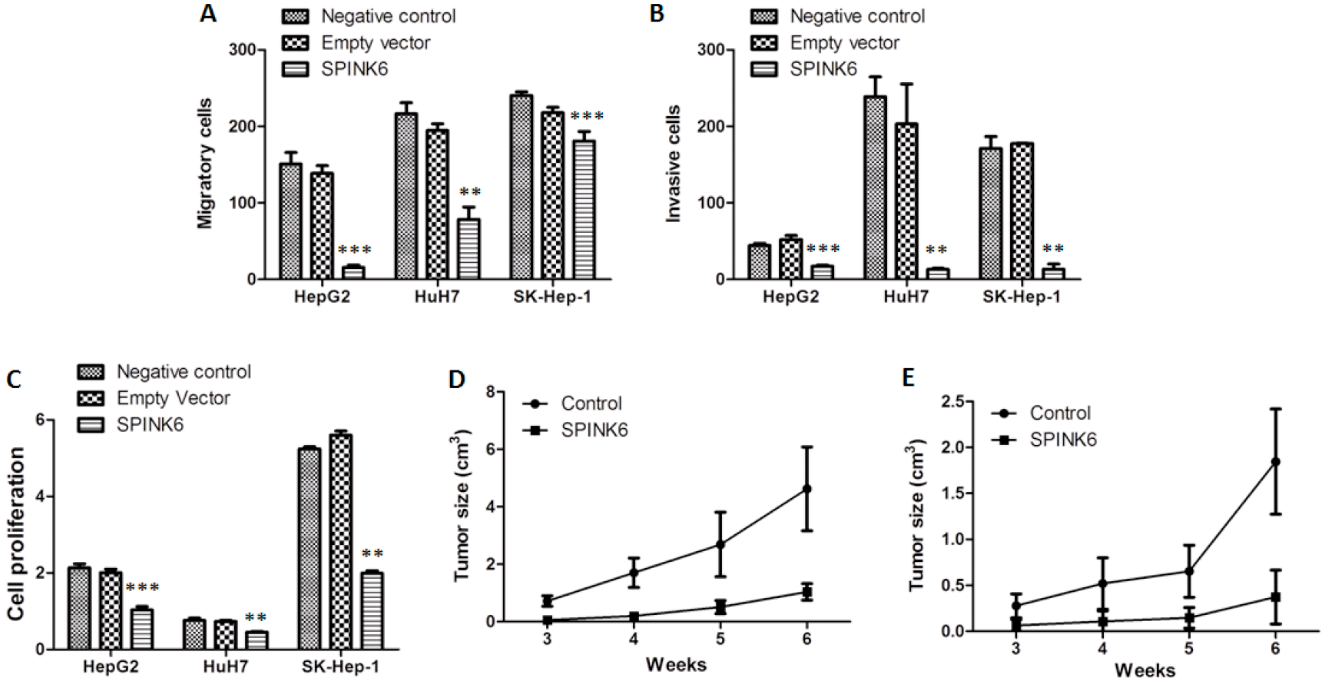

Supplementary Figure S2: SPINK6 suppressed malignancy of different lines of HCC cells. Migration of HepG2, HuH7 and SK-Hep-1 cells with and without SPINK6 overexpression. The same cells were also analyzed for invasion (**B**) and proliferation (**C**) Each assay was repeated 3 times in (**A**) and (B), and 4 times in (C). (**D**) Quantitation of tumor growth after hypodermal injection of HuH7 cells with and without SPINK6 overexpression into nude mice. The cells were injected into 5 nude mice. (**E**) The same quantitation of tumor growth for the SK-Hep-1 cells with and without SPINK6 overexpression. The error bars in all panels are standard deviation. $*P < 0.05$, $**P < 0.01$, $***P < 0.001$.

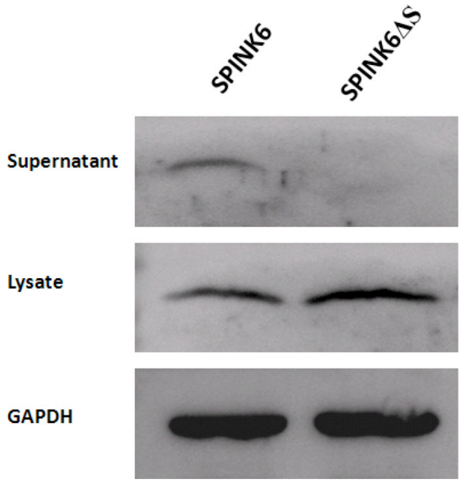

Supplementary Figure S3: Expression of SPINK6 in QGY-7703 cells. Western blot detection of SPINK6 with and without the signal peptide (SPINK6 and SPINK6DS respectively). The western blot was performed against the supernatant (top lanes) of cultured QGY-7703 harboring SPINK6 or SPINK6DS expression plasmids, as well as the lysate (middle lanes) of those cells. GAPDH (bottom lanes) was used as internal controls.

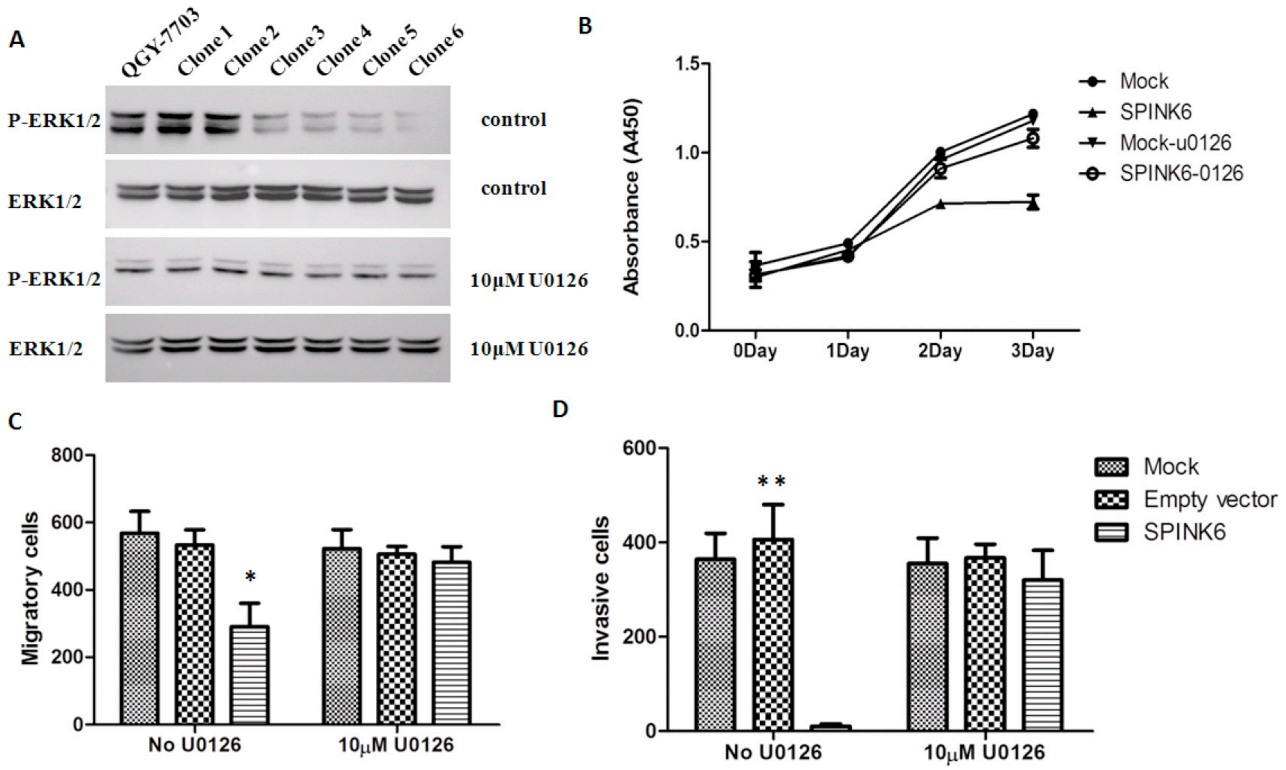

Supplementary Figure S4: The tumor suppression function of SPINK6 was blocked by U0126. (**A**) Western blot analysis of phosphorylated ERK1/2 in QGY-7703 and the derived cell clones expressing increasing amounts of SPINK6. (**B–D**) The proliferation, migration and invasion of QGY-7703 cells. The cells were transfected with a SPINK6 expressing plasmid or an empty vector. Mock represents untransfected QGY-7703 cells. The U0126 compound was added at a 10 μM concentration to block ERK1/2 action. The data points were averaged from 3 assay replicates. Results are expressed as the mean ± SD; *$P < 0.05$, **$P < 0.01$, ***$P < 0.001$.

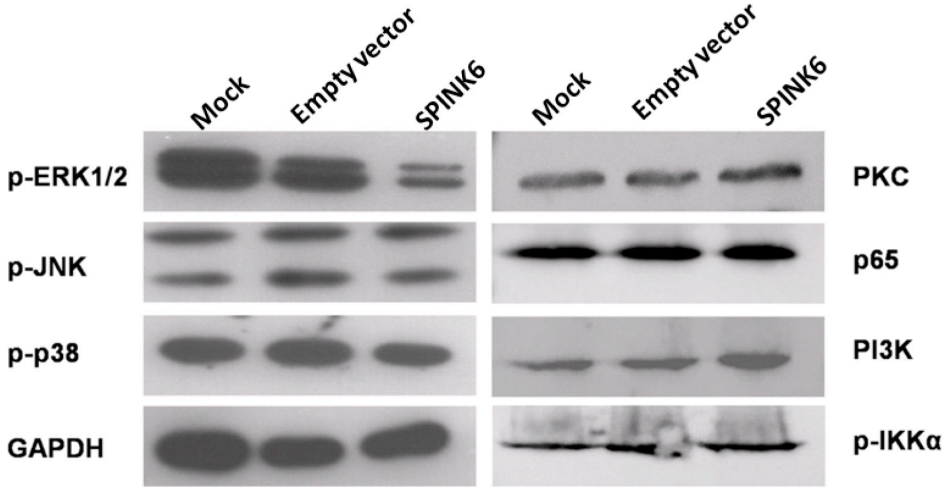

Supplementary Figure S5: Expression and activity of QGY-7703 cell signaling proteins were detected by western blot.

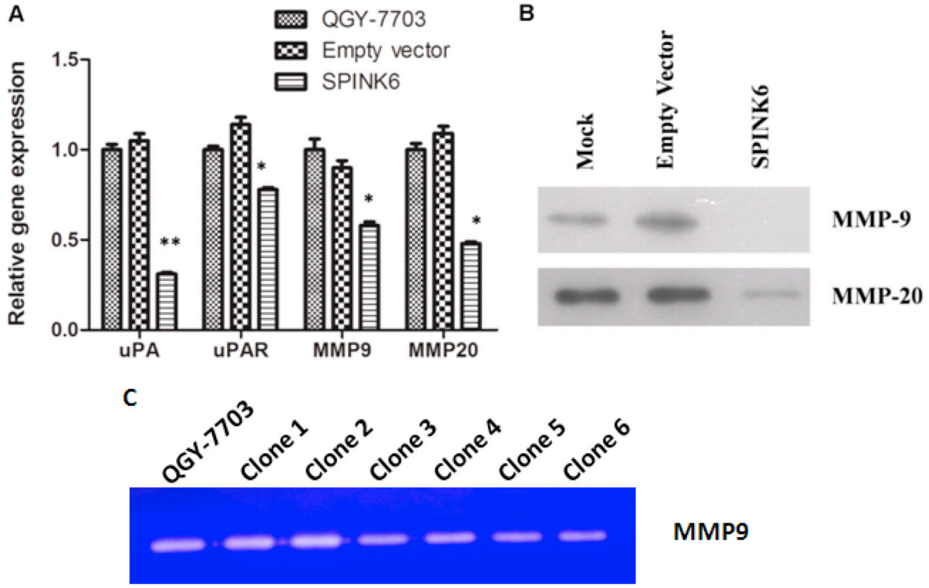

Supplementary Figure S6: (**A**, **B**) Expressions of uPA and MMPs were evaluated by RT-PCR and western blot. (**C**) The biological activity of MMP9 was detected by gelatin zymography.
